# Purposeful Heading Performed by Female Youth Soccer Players Leads to Strain Development in Deep Brain Structures

**DOI:** 10.1089/neur.2021.0014

**Published:** 2021-08-03

**Authors:** Jeffrey S. Brooks, Wayne Allison, Alexandra Harriss, Kewei Bian, Haojie Mao, James P. Dickey

**Affiliations:** ^1^ School of Kinesiology, Faculty of Health Sciences, Western University, London, Ontario, Canada.; ^2^ Health and Rehabilitation Sciences, Faculty of Health Sciences, Western University, London, Ontario, Canada.; ^3^ Department of Mechanical and Materials Engineering, Faculty of Engineering, Western University, London, Ontario, Canada.; ^4^ School of Biomedical Engineering, Western University, London, Ontario, Canada.

**Keywords:** female youth soccer, finite element modeling, linear acceleration, rotational velocity, traumatic brain injury

## Abstract

Head impacts in soccer have been associated with both short- and long-term neurological consequences. Youth players' brains are especially vulnerable given that their brains are still developing, and females are at an increased risk of traumatic brain injury (TBI) compared to males. Approximately 90% of head impacts in soccer occur from purposeful heading. Accordingly, this study assessed the relationship between kinematic variables and brain strain during purposeful headers in female youth soccer players. A convenience sample of 36 youth female soccer players (13.4 [0.9] years of age) from three elite youth soccer teams wore wireless sensors to quantify head impact magnitudes during games. Purposeful heading events were categorized by game scenario (e.g., throw-in, goal kick) for 60 regular season games (20 games per team). A total of 434 purposeful headers were identified. Finite element model simulations were performed to calculate average peak maximum principal strain (APMPS) in the corpus callosum, thalamus, and brainstem on a subset of 110 representative head impacts. Rotational velocity was strongly associated with APMPS in these three regions of the brain (*r* = 0.83–0.87). Linear acceleration was weakly associated with APMPS (*r* = 0.13–0.31). Game scenario did not predict APMPS during soccer games (*p* > 0.05). Results demonstrated considerable APMPS in the corpus callosum (mean = 0.102) and thalamus (mean = 0.083). In addition, the results support the notion that rotational velocity is a better predictor of brain strain than linear acceleration and may be a potential indicator of changes to the brain.

## Introduction

Approximately 90% of head impacts in soccer result from purposeful soccer headers.^1^ Currently, there is no consensus on whether purposeful soccer headers lead to neurocognitive changes. Some studies demonstrate that purposeful soccer heading does not lead to immediate changes in neuropsychological testing^2–4^ or neuroimaging.^4^ In contrast, one study demonstrated a positive association between the number of headers a soccer player performed in a year to the degree of axonal brain injury.^5^ Reduced brain activity^6,7^ as well as increases in radial and axial diffusivity^8^ are demonstrated in adult soccer players. However, interpretations are limited given the cross-sectional study design.^9^

Head impact measurements have been collected to determine purposeful heading kinematics. Soccer game scenario is one factor that influences head impact magnitude during purposeful headers.^10,11^ For example, punts and goal kicks result in significantly larger linear and rotational head accelerations than passes in the air,^11^ whereas passes in the air result in larger linear accelerations than deflections, but smaller linear accelerations than shots. In addition, the linear and rotational head impact kinematics quantified during game scenarios are also influenced by head impact location.^10^ Head impact characteristics affect where the largest brain deformations occur.^12^ Thus, identifying the influence of game scenarios on head impact measurements is an important consideration in determining the potential effects of purposeful headers on the brain.

The majority of soccer studies collecting head impact magnitudes have been on collegiate players.^1,11,13–17^ Some of these studies used video verification for all head impacts whereas others did not, and these studies used a variety of head impact sensors. Accordingly, these studies have reported that purposeful headers result in peak linear and rotational accelerations ranging between 10.0–160.6*g* and 282.8–15,667.5 rad/s^2^, respectively. However, an estimated 22 million youths (persons <18 years of age) play soccer worldwide.^18^ Studies that have examined youth populations have quantified large peak linear and rotational accelerations of 4.5–62.9*g* and 44.8–8869.1 rad/s^2^, respectively, from purposeful headers.^4,10,19,20^ These magnitudes are comparable to those measured in adult populations, yet youth soccer players have reduced head mass and neck strength compared to adults, which may lead to larger head impact accelerations at the same ball velocities.^21–23^

As well, youth brains are still developing and therefore may be more vulnerable to traumatic brain injury (TBI).^24^ Youth players are also more likely to be injured from ball contact as compared to older players.^25^ In particular, 10- to 14-year-old females have a similar concussion incidence as male players^25,26^; however, females have a significantly higher injury rate of head to ball contact than males.^24^ Female soccer has the highest concussion rate among high school female sports and the second highest overall concussion rate (0.35 per 1000 athletic exposures) after American football.^27^ However, limited data exist for female youth soccer populations.

The potential effects of purposeful soccer headers can be determined from brain structure strain measurements. Studies have used finite element modeling to report whole-brain strains in the range of 0.19–0.21 associated with mild TBI (mTBI).^28,29^ However, deformations in specific brain regions cause different symptoms,^30^ highlighting the importance of investigating different brain regions. For example, concussion-related alterations to the corpus callosum result in abnormal interhemispheric functional connectivity and motor impairments.^31^ Some concussion cases are associated with injury to the thalamus and basal ganglia,^32,33^ brain structures that transmit and integrate information.^33^ These brain region–dependent functions have motivated studies to examine the impact response of different brain components such as the corpus callosum, thalamus, and brainstem.^28,29,34–37^ Maximum principal strain (MPS) in gray matter and the corpus callosum are significantly correlated with concussion.^28^ The 50% likelihood of concussion strain threshold varies between the corpus callosum (0.12–0.21),^28,29,34,38^ thalamus (0.12–0.13),^34,38^ and brainstem (0.13–0.19).^29,34,38^

These differences highlight the importance of investigating the effects of impacts on deep brain structures. However, MPSs are sensitive to large strain values sometimes encountered by a small minority of brain elements in brain model simulations.^39^ Accordingly, calculating the average peak MPS (APMPS) for each element in the deep brain structure allows for anatomical specificity and limits the influence of extremely large strain values sometimes encountered in brain model simulations. Measuring strains attributable to purposeful soccer headers can help identify potential dangers for participants. Accordingly, the purpose of this study was to assess the relationship between kinematic variables and maximum principal brain strain during purposeful headers in female youth soccer players. Second, this study sought to compare the APMPS for soccer headers from different game scenarios.

## Methods

### Participants

A convenience sample of 36 female youth soccer players (mean [standard deviation {SD}]; 13.4 [0.9] years of age, 1.6 [0.1] m, 50.6 [8.7] kg) from three elite youth soccer teams (U13, U14, and U15) participating in the Ontario Player Development League (OPDL) were recruited for this study (Table 1). Detailed descriptions of the overall study methodology pertaining to player participation and head impact exposure are reported elsewhere.^6,10,40,41^ Parents provided written informed consent, and players provided written informed assent before participating in the research study. This study was approved by the Health Sciences Research Ethics Board (protocol 107948) at Western University.

**Table 1.** Player and Position Distribution of Female Youth Soccer Players Across Three Age Groups^a^

Position	U13	U14	U15
Defense	4	4	7
Midfield	3	8	1
Forward	3	4	0
**Total**	**10**	**16**	**8**

^a^ Of the 36 recruited players, 1 player never recorded a purposeful header in the season and another player was injured in the first game of the season.

### Instrumentation

Head impacts were recorded using wireless sensors (GForce Tracker; Artaflex Inc., Markham, Ontario, Canada) secured at the back of the head with a headband.^42,43^ A 7 *g* trigger threshold was used, which is consistent with similar studies,^1,44^ given that preliminary data measured before the soccer season indicated that purposeful header impacts can be as low as 8 g. The sensors recorded data from 8 ms preceding the triggered threshold and 32 ms after it. Head impacts were confirmed using video analysis.^45,46^

### Impact selection

Data were collected for one entire season (60 regular season games, 20 games per team). A set of 434 purposeful headers were identified and categorized by game scenario (Table 2). An aggregate impact magnitude score was calculated for each of the purposeful headers based on the percentiles of the peak linear acceleration and angular velocities. A representative set of 110 impacts was identified using this aggregate score, including the maximum and minimum cases.

**Table 2.** Definition of the Game Scenarios for Purposeful Headers in Soccer

Header context	Description of game scenario
Corner kick	Header after a stationary kick from the corner of the field
Deflection	Header after the ball contacts another player or body location
Punt^a^	Header after a kick in which the goalie drops the ball from their hands and kicks it before it impacts the ground
Goal kick	Header after a kick of a stationary ball awarded to one team after the ball crossed the goal line by the attacking team
Pass in air	Header of a ball that was kicked in the air
Free kick	Header after a kick of a stationary ball after one team commits a foul
Throw-in	Header after a player throwing the ball into play from the sideline

^a^ Also called a drop kick by some researchers.

### Finite element modeling

The Global Human Body Models Consortium (GHBMC) head model was used for the finite element model simulations.^47^ This model has been validated against 35 experimental cases.^47^ It contains elements representing the skin, skull and facial bones, sinuses, cerebrum, cerebellum, lateral ventricles, corpus callosum, thalamus, and brainstem as well as white matter. This head model is based on the simulation's kinematic boundaries and therefore indirectly incorporates neck musculature contributions.

There is no validated youth head model; therefore, the GHBMC model was scaled to linear dimensions of 95.83% (88% of the volume), representing a 13-year-old female^48^ and was determined from head volume differences between sexes.^19,48–53^ Specific details are presented in the primary GHBMC description.^47^

A commercially available software (LS-DYNA; Livermore Software Technology Co., Livermore, CA) was used to run the simulations with an 8-core processer. The simulation boundary conditions were defined according to the time-series data collected by the wireless sensors for each head impact case. Specifically, the linear acceleration and angular velocity data collected for each purposeful header determined the kinematics of the head center of mass, similarly to previous research.^35,39,54^

Three specific brain regions were selected for analysis based on previous imaging research highlighting regions affected during mTBI.^55–58^ The corpus callosum was selected because imaging revealed disruptions in development in children with concussions^59,60^ as well as soccer players experiencing repetitive head impacts.^8^ The thalamus was selected because it has vast connections throughout the brain and makes key contributions to the integration and assimilation of sensory and motor information.^61^ Third, the brainstem was selected because of its involvement in motor activity^62^ and the observation that standing balance is often impaired in concussed athletes.^63^ Specific regions of interest (ROIs) were identified based on their anatomical location in the finite element mesh. Within each ROI, the MPS in the elements was calculated using LS-PrePost. The peak maximum principal strain of each element over the time series from each ROI was extracted. These values were averaged to yield a peak MPS value for the entire ROI, the APMPS.

### Statistical analysis

Chi-squared analyses were performed to evaluate whether the proportion of head impacts for the different age groups, and game scenarios, in the sample of 110 simulations was representative of the full set of head impacts. A Shapiro-Wilks normality test determined the normality of the distribution of the linear acceleration and angular velocity data. In the event of failed normality tests, medians and interquartile ranges are reported for the impact parameters.

All statistical analyses were performed in R (R Foundation for Statistical Computing, Vienna, Austria),^64^ with linear mixed-effects analyses conducted using the lme4^65^ and lmerTest^66^ packages. A linear mixed-effects model determined whether game scenario, brain region (corpus callosum, thalamus, and brainstem), linear acceleration, and angular velocity predicted brain strain. Fixed effects included game scenario (Table 2), brain region, linear acceleration, and rotational velocity. Random effects included individual differences and player age. Statistical significance was defined using a 0.05 threshold. Linear mixed-effect modeling effect sizes were not calculated because they can be misleading and inaccurate.^65^

Pearson product-moment correlations were calculated to evaluate the strength of the relationship between APMPS and peak angular velocity, and between APMPS and peak linear acceleration within each ROI. Correlation coefficient (*r*) values between 0.0 and 0.3 were considered negligible, 0.3–0.5 low, 0.5–0.7 moderate, 0.7–0.9 high, and 0.9–1.0 very high.^67^

## Results

Both age (χ^2^ = 1.89, *p* = 0.38) and game scenario (χ^2^ = 3.98, *p* = 0.68) were not significantly different between the subset and the total data set, reflecting that the subset of headers was representative of the total data set.

The median (interquartile range) linear acceleration for the purposeful headers was 16.1*g* (12.3–22.1), with a maximum of 74.8*g*. The median angular velocity was 16.2 rad/s (10.5–21.2), with a maximum of 50.6 rad/s. Of the 110 head impacts, 76 were located at the front of the head, 31 at the top of the head, and three at the side of the head (Table 3).

**Table 3.** Number of Headers for the Different Game Scenarios for the Full Set (434 Headers) and Subset (110 Headers), and the Associated Head Impact Kinematic Data

Game scenario	Total no. (%) verified by video	Number (%) in FE analysis	Median (IQR) linear acceleration (*g*)	Median (IQR) angular velocity (rad/s)
Corner kick	12 (2.8)	3 (2.7)	31.4 (27.9–32.0)	39.6 (30.2–40.0)
Deflection	43 (9.9)	10 (9.1)	9.9 (8.8–17.5)	9.5 (6.3–14.0)
Punt	35 (8.1)	7 (6.4)	13.4 (11.2–15.8)	12.7 (11.0–19.6)
Goal kick	16 (3.7)	2 (1.8)	24.8 (21.4–28.3)	22.4 (21.7–23.1)
Pass in air	179 (41.2)	47 (42.7)	16.2 (12.7–24.7)	19.1 (14.8–23.8)
Free kick	20 (4.6)	2 (1.8)	22.9 (17.2–28.6)	18.9 (15.1–22.8)
Throw-in	129 (29.7)	39 (35.5)	16.0 (12.5–19.0)	13.7 (9.4–18.4)
**Total**	**434 (100)**	**110 (100)**	**16.1 (12.3–22.1)**	**16.2 (10.5–21.2)**

FE, finite element; IQR, interquartile range.

Within the deep brain regions assessed, the APMPSs observed for the corpus callosum, thalamus, and brainstem were 0.102 (95% confidence interval [CI] = 0.085, 0.119), 0.083 (95% CI = 0.069, 0.097), and 0.048 (95% CI = 0.040, 0.056), respectively.

**FIG. 1.**
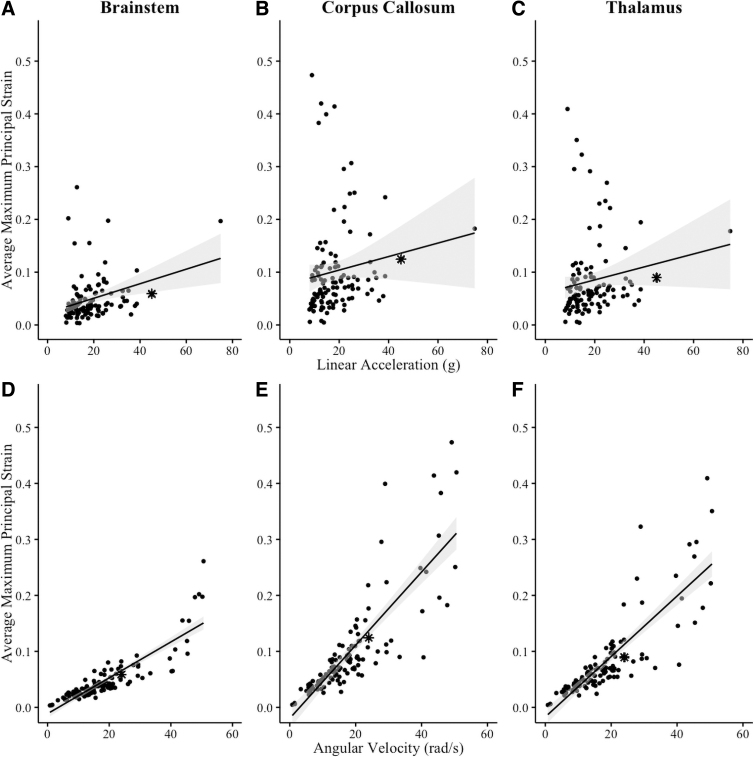
Relationship between average maximum principal strain and kinematic variables in the brain for youth female soccer players' purposeful headers. Shaded area represents 95% confidence interval. Linear acceleration (*g*) is on the top, and angular velocity (rad/s) is on the bottom. Concussion case indicated with an asterisk (*).

In the brainstem, there was a weak positive association between the APMPS and linear acceleration (*r* = 0.31, *p* < 0.001) and a high positive association between the APMPS and rotational velocity (*r* = 0.87, *p* < 0.001). In the corpus callosum, there was a negligible association between the APMPS and linear acceleration (*r* = 0.13, *p* = 0.17) and a high positive association between the APMPS and rotational velocity (*r* = 0.83, *p* < 0.001) in the corpus callosum. Last, in the thalamus, there was a negligible positive association between the APMPS and linear acceleration (*r* = 0.16, *p* = 0.10) and a high positive association between the APMPS and rotational velocity (*r* = 0.84, *p* < 0.001; Fig. 1).

The majority of purposeful headers occurred from passes in the air, whereas purposeful headers from free kicks and goal kicks occurred the least. Purposeful headers that occurred from corner kicks had the largest median magnitudes for both linear acceleration and angular velocity (Table 3). Purposeful headers that resulted from throw-ins, punts, and deflections fell below the total median for linear acceleration and angular velocity.

Angular velocity (*F*_1_ = 264.78, *p* < 0.001) was a statistically significant predictor of APMPS in the brainstem, whereas game scenario (*F*_6_ = 1.39, *p* = 0.23) and linear acceleration (*F*_1_ = 0.51, *p* = 0.48) were not. Linear acceleration (*F*_1_ = 17.62, *p* < 0.001) and angular velocity (*F*_1_ = 227.17, *p* < 0.001) were statistically significant predictors of APMPS in the corpus callosum, whereas game scenario was not (*F*_6_ = 0.41, *p* = 0.87). Linear acceleration (*F*_1_ = 16.05, *p* < 0.001) and angular velocity (*F*_6_ = 233.22, *p* < 0.001) were statistically significant predictors of APMPS in the thalamus, whereas game scenario was not (*F*_6_ = 0.43, *p* = 0.86).

**FIG. 2.**
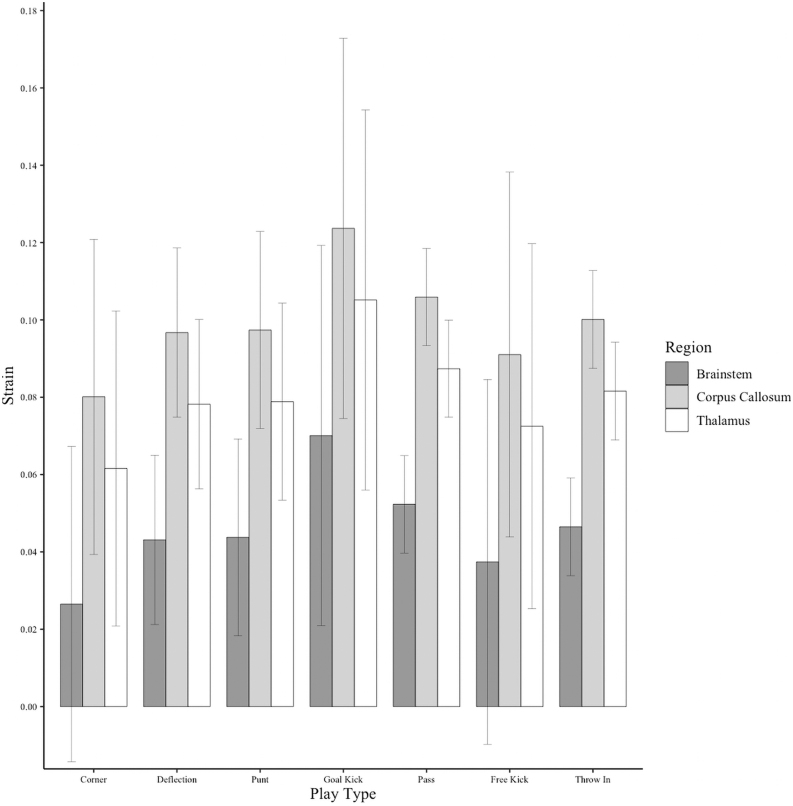
Bar graph of female soccer brain strains in the brainstem, corpus callosum, and thalamus form purposeful headers during different plays. Error bars indicate 95% confidence intervals.

APMPS values were highest in the corpus callosum and lowest in the brainstem across all game scenarios. Goal kicks induced the highest APMPS, whereas corner kicks induced the lowest (Fig. 2).

## Discussion

Our analysis indicated that game scenario was not a contributing factor to predicting APMPS of purposeful headers. This may be attributable to the fact that impact location causes different strains in various brain regions,^30,68^ but the majority of head impacts in this study (70%) occurred at the frontal location across all game scenarios.

In this study, game scenario was not a predictor of APMPS; however, rotational velocity was strongly associated with APMPS in all three regions of the brain during purposeful headers whereas linear acceleration was poorly associated with APMPS (Fig. 1). Other finite element head-model studies have measured head kinematics and the associated brain responses with similar conclusions that rotational velocity best predicted MPS^69^ or strongly correlated with MPS.^70,71^ Previous research has indicated that linear acceleration is a good indicator of intracranial pressure, but does not indicate brain deformations as well as rotational kinematics.^28^ Our analysis supports this notion, indicating that rotational velocity measurements could be used to monitor potential brain strain in youth soccer players in real time.

Previous studies have found that the MPS values of these brain structures range in various sports. Particularly, in the corpus callosum, the MPS for concussive impacts in Australian rules football, American football, and hockey ranged from 0.28 to 0.31,^34,36^ which exceeds the threshold for 50% likelihood of concussion (0.12–0.21).^28,29,34,38^ For the thalamus, concussive impacts in Australian rules football and American football measured an MPS ranging from 0.26 to 0.38,^34,37^ also exceeding the threshold for 50% likelihood of concussion (0.12–0.13).^34,38^ In the brainstem, MPSs of 0.21 have been measured in Australian rules football concussive impacts, exceeding the 50% likelihood of concussion (0.13–0.14).^34,38^ Whereas this study focused on average strain for the entire ROI and calculated average MPSs that were below the aforementioned values, the median strain of 0.12 for the corpus callosum during headers such as goal kicks (Fig. 2) indicated that significant strain levels developed inside the deep brain. Long-term consequences of high APMPS values are not known; however, a previous study of this same player cohort used cumulative purposeful headers to successfully predict subclinical changes in electroencephalogram power in some frequency bands.^6^

There are some limitations to the current study that should be acknowledged. Only purposeful headers for OPDL female youth soccer players were measured; therefore, we cannot comment on whether other soccer leagues or calibers would show similar purposeful heading exposures. A previous analysis of these data reported that player position did not have a statistically significant effect on the number of purposeful headers a player performed, so it was not considered in this article.^41^ Exposure to repetitive head impacts may cause greater brain tissue injury in female soccer players compared to males^72^; accordingly, these findings may not be applicable to male soccer players given that males head the ball more frequently.^4^ Further, our heading data only includes games and not practices; therefore, we cannot comment on any magnitude or frequency differences in heading behaviors between games and practices that may influence brain strains. Last, only purposeful headers were measured, whereas unintentional head impacts can lead to larger head impact accelerations.^13^

The impact magnitudes in this study are based on sensor recordings attached to the back of the head rather than predicted head center of mass accelerations. The sensors record for 40 ms during the impact, which is appropriate for measuring peak accelerations and has been previously used in soccer studies.^19^ Accordingly, finite element model simulations have been performed using a 40-ms window length.^73^ However, some brain-strain measurements in this data set were still increasing at 40 ms. Thus, the data in this study represent the peak strains recorded within the 40-ms simulation window, but larger peak strains may have occurred beyond this time frame.

The results of this article are specific to the scaled GHBMC model of the brain that was used. The model depends on material properties and characteristics of cadavers and may be different to those of a living human. However, the GHBMC model has been validated against experimental cases.^47^ The ROIs in this research were the corpus callosum, brainstem, and thalamus. Although these brain structures have been identified as areas impacted by concussion, other brain areas may also be affected by this type of injury.

MPSs are sensitive to local changes in geometry and material properties and are not directional. We addressed this volatility by calculating average strains over all elements in the ROI.^71^ Brain tissues that have a specific orientation (such as white fiber tracts) may not be best described using MPS magnitudes. The time-consuming nature of the reconstruction process restricted the number of cases that were simulated. Accordingly, the analyzed cases were selected to represent the full data set.

Our analysis indicates that purposeful heading in female youth soccer players induces strain development on deep brain structures. This strain was strongly associated with head rotational velocity measurements during the event. This information can be used by coaches and league administrators alike in developing safer heading protocols for Canadian youth players. The U.S. Soccer Federation limited the number of purposeful headers that youth players perform, banning heading for players younger than 10 years of age and limiting the practice of heading for those 11–13 years of age.^74^ Reducing the size, weight, and pressure of the soccer ball reduces linear accelerations and rotational velocities in youth players,^75^ which could be an alternative approach to controlling cumulative heading burden. Therefore, the results of this study indicate a critical need to develop policies and protocols that help protect subcortical tissue of developing female brains, which are at an increased risk of injury due to purposeful soccer heading.

## Conclusion

We measured head linear accelerations and rotational velocities for a total of 434 headers from 36 female youth players during soccer games and conducted 110 representative simulations investigating deep brain strain during these headers. Results demonstrated considerable APMPSs in the corpus callosum with a mean of 0.102 and in the thalamus with a mean of 0.083. Rotational velocity was strongly associated with APMPS in all three regions of the brain during purposeful headers. Linear acceleration was poorly associated with APMPS. Game scenario was not an independent contributing factor to predicting APMPS during female youth soccer. Future studies should incorporate rotational velocity measurements as an indicator for brain injury for female youth soccer players.

## Abbreviations Used

APMPS: average peak maximum principal strain
CI: confidence interval
GHBMC: Global Human Body Models Consortium
MPS: maximum principal strain
mTBI: mild traumatic brain injury
OPDL: Ontario Player Development League
ROIs: regions of interest
TBI: traumatic brain injury
